# Industrial Investigations of S355 Steel-Grade Homogenization in a 100-Tonne Ladle Furnace

**DOI:** 10.3390/ma18010180

**Published:** 2025-01-03

**Authors:** Dariusz Jochymczyk, Marek Warzecha, Artur Hutny

**Affiliations:** 1 LIBERTY Częstochowa sp. z o.o., ul. Kucelińska 22, 42-207 Częstochowa, Poland; 2 Faculty of Production Engineering and Materials Technology, Częstochowa University of Technology, al. Armii Krajowej 19, 42-201 Częstochowa, Poland; artur.hutny@pcz.pl

**Keywords:** ladle furnace, ladle, numerical modelling, steel homogenization

## Abstract

The paper presents the results of industrial research and numerical simulations of the chemical homogenization of liquid steel. The research object was a ladle furnace with a working capacity of the ladle of 100 t at the steel plant of Huta Częstochowa, currently Liberty Częstochowa Sp. z o.o. Industrial research was carried out under standard production conditions of the steelworks. The research included automatic steel sampling, measurement of the bath temperature, controlled measurement of argon flow at a given intensity, and the determination of the concentration of elements in steel samples using a spectrometric analyser. The element introduced in the form of a ferroalloy (FeMn and FeSiMn) played the role of a marker in the study of changes in the chemical composition during the process of dissolution and mixing of the alloying additive. Monitoring changes in the chemical composition of steel after the introduction of the marker was carried out by taking metal samples. The initial and boundary parameters of the modelled processes necessary to perform numerical simulations were determined successively through industrial measurements or determined on the basis of empirical relationships. A two-equation k-ε turbulence model was used to assess the flow inside the tested ladle furnace, and a discrete phase model was used to model gas bubbles. The mixing characteristics of the steel bath after introducing the alloying additive to it were determined. The comparison of the results of numerical simulations with experimental data was based on the analysis of the chemical homogenization process.

## 1. Introduction

Contemporary steel metallurgy, thanks to refined and continuously improved technological processes, offers the possibility of obtaining products of increasingly higher quality. The nature of the refining processes has resulted in the transfer of its main technological operations outside a steel melting furnace, hence the introduction of the concept of secondary metallurgy. The combination of melting processes in the furnace (whether in a basic oxygen furnace or an electric arc furnace, EAF) and subsequent secondary metallurgy (mainly ladle furnace, LF) forms an integral part of steel production technology. Nowadays, there is still a continuous development of these processes in terms of quality and economy. Clients are placing increasingly higher demands on the quality of steel. As a result, high-purity steels are now being produced that depend on the content of harmful admixtures, chemical and phase segregation, and non-metallic inclusions [1,2,3,4,5]. Quality improvements are being achieved through, among other things, higher homogenization and lower contamination levels in steel, consistency, and a narrow range of chemical composition. Economic improvements are also being realised by achieving higher yields of alloying additives introduced into the steel and eliminating “missed” heat options. In recent years, the field of model-based research on steelmaking processes has developed very intensively. Modelling research initially evolved as physical (water) models, and later—thanks to a significant increase in the computational power of high-performance computers—mathematical models, utilising computational fluid dynamics (CFD), emerged [6,7,8]. While these models are often used as support for researchers, they have their simplifications and limitations. In the case of water models, where molten steel is replaced by water, the focus is on the scale of metallurgical aggregates or flow similarity [9]. In numerical models, the focus is on the complexity and simplifications, such as turbulence models or the omission of the slag phase. Therefore, conducting experimental research on an industrial device is crucial, as it not only serves as the most valuable source of research information but also as a verification tool for supporting model-based studies. The ongoing research is highly relevant and important, as evidenced by the publications from research centres worldwide [10,11,12].

The issues presented in this work, planned to be realised as industrial research, were carried out at the ISD Huta Częstochowa production plant (currently LIBERTY Częstochowa), while model research was conducted at the Chair of Metals Extraction and Recycling at the Faculty of Production Engineering and Materials Technology of the Częstochowa University of Technology.

The paper presents the results of research into the homogenization of S355 steel. The ongoing research is highly relevant and important, as evidenced by the publications from research centres worldwide. These studies primarily aim to optimise steelmaking processes and minimise their negative impact on the environment by reducing the consumption of various media and limiting emissions. Such research enables the influence of specific process variables and the recording of system interactions, determining their impact on the entire, complex process. This allows the identification of optimal parameters and process variables. As part of the research, the characteristics of the steel mixing process following the introduction of an alloying additive were developed under industrial conditions. The necessary time to achieve the desired chemical and thermal homogenization was determined. The added value of the conducted research is a series of industrial studies, which provide information on the complex process of alloy dispersion in liquid steel. Industrial studies are particularly valuable because, unlike model studies, we are not dealing with a marker, but with a complex alloy additive, which was a binary alloy (FeMn) and a ternary alloy (FeSiMn). The research results make it possible to modify the ladle furnace control software, and in particular to optimise the inert gas blowing time, depending on the amount and type of alloy additive.

## 2. Experimental Procedure

The object of the industrial research was a ladle furnace (LF) at the steel plant of Huta Częstochowa steelworks (currently Liberty Częstochowa Sp. z o.o.). This furnace was built by the company “ELKEM SKANLANCE”—Sweden. The ladle furnace consists of a steelmaking ladle adapted for the preparation of the appropriate steel grade for sequential casting on the continuous casting machine (CCM) and for the process of heating the liquid steel with an electric arc. The steelmaking ladle with the liquid metal, after the base steel is tapped from the “Danieli” electric furnace, is transferred to a ladle transfer vehicle designed for ladle transport and then, via the ladle transfer vehicle, to the ladle treatment station. The working volume of the ladle is adapted to the capacity of the electric furnace and is approximately 100 tonnes of liquid steel.

At the steel plant, liquid metal is melted from steel scrap and pig iron in an electric arc furnace—Danieli—Italy (ultrahigh power, UHP).

During the industrial research, the furnace operated under standard production conditions, i.e., melted steel quantity of about 100 tonnes, and input material: scrap and additional solid pig iron (in the amount of 14–17% of the total input weight).

The carbon content in the steel leaving the furnace is about 0.02–0.10% lower than the maximum allowable carbon content in the finished steel. The steel-tapping temperature from the furnace ranges from 1630 to 1690 °C.

The steel is tapped from the furnace without slag. During tapping, desulphurisation treatment, aluminium deoxidation, and the initial addition of silicon and manganese to the steel (70–80% of the target content for a given grade) are carried out. The tapping process takes up to 3 min. In the final phase of tapping, the furnace slag is cut off based on a visual assessment of the steel quantity in the ladle. The separation of slag from metal is facilitated by the location of the tapping port in the furnace, which allows metal to flow out first during the tilting of the furnace on the so-called “cradle”. If some slag enters the ladle during the final phase of tapping, it can be removed using a device called a “slag skimmer” if necessary, and the ladle with the metal is then transported to the appropriate station.

Before commencing treatment at the LF station, synthetic slag with a composition of approximately 75% CaO + 25% Al_2_O_3_ and granulated aluminium for slag deoxidation are introduced.

Secondary steelmaking is carried out in the ladle furnace. During this process, the steel is refined, and a desulphurisation process takes place to correct the sulphur content and to achieve the assumed chemical composition for the produced steel grade. Throughout the secondary metallurgy process, the steel is almost continuously purged with argon. Additionally, the ladle furnace has the capability to purge the steel with argon using a lance.

Basic data of the ladle furnace are presented in Table 1.

The steelmaking ladle is equipped with two gas-permeable fittings built into the ladle bottom (an off-centre fitting, referred to as K1, and another fitting, K2, located near the centre of the ladle bottom). Additionally, the ladle lining in the slag zone is designed to be suitable for electric arc heating. The ladle is equipped with an argon delivery system to the fitting(s), along with an automatic connection to the vacuum degassing device—a VD station. In addition to the porous fittings embedded in the bottom of the ladle, the ladle furnace is equipped with devices for introducing powdered CaO using a lance or in the form of a flux-cored wire.

The ladle lining in the steel zone consists of a magnesite layer, while in the slag zone, it is a carbon-magnesite layer. This lining typically lasts for about 25–30 heat options.

**Figure 1:**
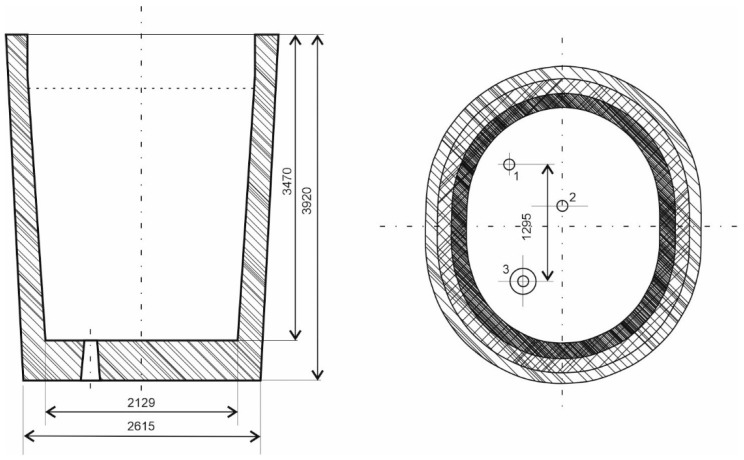
The steelmaking ladle involved in the industrial research, 1, 2—Ar plugs; 3—ladle nozzle.

Figure 1 depicts a schematic diagram of a steelmaking ladle, along with its key dimensions, adapted for ladle treatment and a treatment station with the capability of heating by electric arc energy.

All the final parameters of the liquid steel of a given processed grade are obtained at the ladle furnace station. In order to achieve the correct chemical composition of the steel, an important technological operation is carried out, which accelerates processes such as the homogenization of the liquid steel throughout its volume or the process of homogenization of the chemical composition after the introduction of an alloying additive. This operation is the mixing of liquid steel by means of the injection of inert gas (Ar), introduced into the ladle by means of a special gas-permeable fitting embedded into the bottom of the ladle. An important parameter of this technological operation is the determination of the gas flow rate, supplied appropriately for a given object, in this case for a given ladle geometry. Determining the so-called optimum flow rate, which affects the time required for complete mixing of the liquid steel, is very important and was one of the significant intermediate objectives of this research.

**Figure 2:**
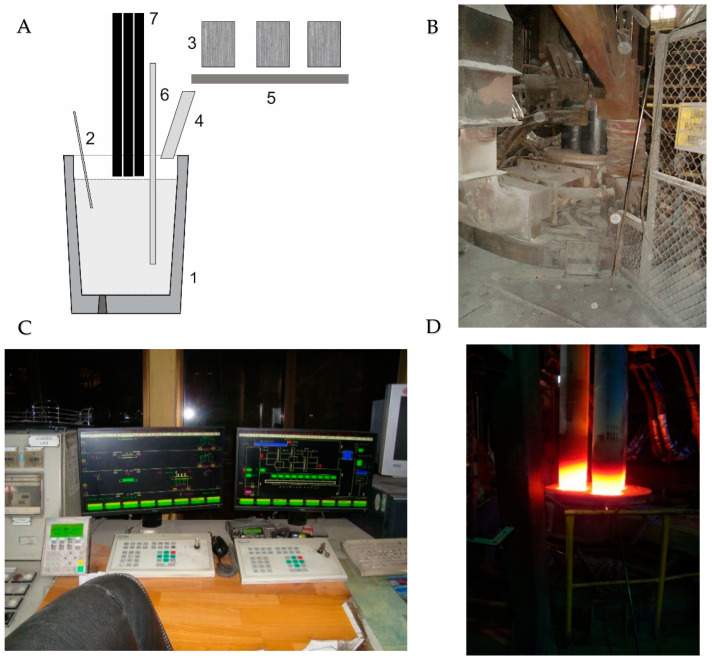
Ladle furnace (LF) station: (**A**) schematic diagram and view of station with control room, 1—steelmaking ladle, 2—automatic temperature measurement and sampling of metal and slag, 3—ferroalloy hoppers, 4—feeding trough, 5—automatic scale, 6—lance for injecting CaO; 7—graphite electrodes; (**B**) view of top of roof; (**C**) control room; (**D**) graphite electrodes.

The LF station is equipped with automated devices for weighing and dosing slag-forming and alloying additives, automatic steel sampling—integrated with a device for measuring oxygen activity and bath temperature, regulated argon flow at a preset rate, and determining the concentration of 20 preset elements in steel samples using an integrated, containerised spectrometric analyser. A schematic diagram and photographs of the test stand are shown in Figure 2.

Industrial research was carried out during the production of low-alloy steels; the technological operations carried out in the ladle furnace during steel processing are listed below:

- Argon injection with an adjustable flow rate: The flow rate can be adjusted within the range of 50 to 600 dm^3^/min.
- Electric arc heating with adjustable arc power: Heating power is regulated by heating elements, with the furnace’s maximum power being 30 MW.
- Measurement of liquid steel temperature using a disposable immersion sensor: Measurement is carried out with a “CELOX” device.
- A chemical analysis of liquid steel by means of metal sampling and measurement in a containerised mass spectrometer.
- Weighing and transfer equipment for feeding ferroalloy additives into the ladle: This involves a set of weighing feeders dispensing suitable materials onto a conveyor belt that drops the measured ferroalloys into the ladle onto the metal’s surface exposed by the argon process.
- Equipment—flux-cored wire feeders: The flux-cored wires are mainly used to feed the materials that modify inclusions remaining after the deoxidation of liquid steel with aluminium. This low-density material, when fed in this way, can dissolve in the metal volume near the bottom of the ladle. Sometimes rare earth metals are also fed using this method.
- Weighing equipment for feeding synthetic slag components: It enables the precise measurement of synthetic slag with the appropriate composition. The standard synthetic slag fed at the LF station is primarily composed of CaO (up to 80%) and a liquefier in the form of bauxite Al_2_O_3_ (about 20%). Feeding occurs via a spreader, onto the surface of the ladle, before it is transported on the ladle transfer vehicle to the ladle furnace treatment station.

The industrial research was conducted under the steel mill’s standard production conditions. Measurements were taken using standard measuring equipment to determine the average conditions of the steel manufacturing process for selected grades.

The research involved the following:

- Automatic steel sampling;
- Bath temperature measurement;
- Controlled argon flow measurement at a preset rate;
- The determination of element concentrations in steel samples using a containerised spectrometric analyser.

The industrial research was carried out during the production of S355-grade steel. The chemical composition of the steel is provided in Table 2.

In the first part of the research, the determinations concern the changes in the temperature and chemical composition of the steel during the dissolution and mixing process of the alloying additive—after its introduction into the liquid steel. The role of the marker was played by the element introduced in the form of a ferroalloy (FeMn and FeSiMn), with compositions provided in Table 3.

The monitoring of changes in the chemical composition of the steel after the introduction of the marker was carried out by taking metal samples, colloquially known as “lollipop” or “soap bar” samples, at a specified frequency of about 3 samples per minute, depending on the technical capabilities during a specific industrial experiment. The number of samples varied depending on parameters such as the mass of the introduced alloying additive, the flow rate of the injected argon, and the duration of the process. The steel plant is equipped with a laboratory spark spectrometer with a vacuum optical chamber for the precise analysis of the composition of low-alloy, stainless, chrome, chrome-nickel, free-machining, and tool steels, starting from concentrations expressed in ppm (parts per million). The spectrometer also has the capability to measure nitrogen content in the steel.

Additionally, the plant is equipped with a Celox-Lab meter from Heraeus Electro-Nite, coupled with an automatic measurement device, enabling the measurement of temperature and oxygen activity in liquid metal using disposable sensors installed on a special measuring lance. Key features of this instrument include accuracy and repeatability of results with a short measurement time (several seconds). The initial and boundary parameters necessary for conducting numerical simulations of the modelled processes were determined progressively through industrial measurements (Table 4) or derived from empirical relationships. Thus, for example, the density of liquid steel was calculated from an empirical relationship based on industrial measurement data.

The density of the liquid steel was determined from a relationship taking into account the temperature and carbon content of the liquid steel [13]:

(1) $$\rho_{s}=\frac{8319.49-0.835\cdot T}{1-0.01\cdot C}$$

where ρ_s_ is the density of liquid steel, kg/m^3^; T is the temperature, °C; and C is the carbon content of the steel, in % _mass_.

The density values of liquid steel for the given steel grades, calculated according to Equation (1), are presented in Table 5.

The other physico-chemical parameters of the liquid steel adopted for the numerical calculations are listed in Table 6.

Based on the industrial determinations (Table 7), it can be inferred that—excluding a few heat options in the LF where the time was extraordinarily extended (over 90 min) due to technological reasons—the average treatment time in the LF is about 34 min, with an average heat mass of approximately 103 tonnes of liquid steel.

The argon flow rate is extremely important for chemical homogenization time. Table 7 also presents the average argon flow per heat, which is derived from the treatment time and the total gas expenditure per heat. Excluding the heat options where the holding time of the ladle at the LF station is exceptionally long, groups of heat options can be identified where the argon flow rate is around 300, 400, and 500 dm^3^/min, respectively. When considering the minimum argon flow rate through the fitting, it is important to note that above the non-centrically located fitting (main fitting, K1), there is a chute for introducing alloying additives into the metal bath. Therefore, the minimum gas flow rate through this fitting must ensure the exposure of the metal surface from the covering slag layer, which is crucial when introducing alloying additives into the steel. For the ladle under consideration, the level of such a minimum flow is about 300 dm^3^/min. In industrial conditions, there are occasional instances where argon is introduced at levels of 600 dm^3^/min or even exceeding 700 dm^3^/min. Such conditions are often caused by leakage in the argon installation near the ladle (due to high temperatures affecting various connections). The actual physical argon flow through the fitting is then lower than intended for the given process. Such high gas flow rates, if introduced, can lead to disruptions in the interfacial surface between the liquid metal and slag, and even cause situations where metal and slag are expelled over the ladle edge and outside, potentially leading to equipment failure. The disruption of the interfacial surface will result in the mixing of the slag/metal phases, which in turn will cause slag droplets to enter the liquid steel, thereby reducing its purity. In order to avoid this, it is necessary to determine the maximum argon injection rate through the fitting at which slag/metal mixing does not yet occur. Kim et al. [16] proposed an empirical formula to determine the maximum argon flow rate through the gas-permeable fitting that prevents the mixing of slag and metal phases:

(2) $$Q_{cr}=0.067\cdot H^{1.81}{\left(\frac{\sigma \Delta \rho }{\rho_{s}}\right)}^{0.35}$$

where Q_cr_ is the critical argon flow rate, dm^3^/s; H is the height of the analysed ladle, cm; σ is the surface tension, dyne/cm; Δρ is the density difference between liquid steel and slag, g/cm^3^; and ρ_s_ is the density of liquid steel, g/cm^3^.

The values presented in Table 8 determine the maximum critical argon flow rate for the studied steelmaking ladle, considering various extreme variables: interfacial tension between steel and slag, steel density, and slag density. The calculated values set the permissible argon flow rate for the studied ladle at a level not exceeding 600 dm^3^/min. These indications strongly suggest that for the studied ladle, it is appropriate to use argon injection rates ranging from 300 to 500 dm^3^/min.

During the production of the steel under study, the standard alloying additive is FeMn, which is added in varying amounts depending on the requirements. The chemical composition of the two groups of structural steels involved in the study, S235 and S355, mainly differs in the content of Mn, C, and Si. These are the two primary steel grades produced at the plant, categorised according to the needs of the client and the resulting strength properties into so-called grade codes. The chemical composition of the grade codes is consistent for a given grade, but the content of a specific element may fall within a certain range to appropriately emphasise certain chemical or strength properties of that code. As a result, individual grade codes contain varying but similar amounts of Mn, which always fall within the range defined for the grade. This leads to similar amounts of alloying additives being used for the produced grades for specific codes.

During production, ferroalloys are added in two stages. In the first stage, the additives in the form of ferroalloys are added to the ladle into the metal stream during tapping. The second stage involves adjusting the chemical composition at the LF station and partially at the VD station. In the first stage, during the tapping of the base steel, aluminium chunks are added as a deoxidiser, along with ferroalloys in quantities dependent on the produced grade code. These basic ferroalloys are FeSiMn and FeSi in an amount of approximately 80% for a given grade code. The additives in the form of FeSiMn and FeSi, together with aluminium, act as a deoxidiser, and therefore their dross varies depending on the overoxidation of the bath (base steel from the furnace). As a result, different amounts of these elements are present in the ladle after tapping. In the second stage, after the thermal and chemical homogenization of the steel in the ladle at the ladle furnace station, a metal sample is taken for the chemical analysis, and the composition is further adjusted for the specific grade code. During treatment at the LF station, the metal bath is preliminarily deoxidised with additives at the metal tapping station, resulting in a low dross of ferroalloys added at the LF station. Ferroalloys are added depending on the required chemical composition of the processed grade code.

From Table 9, it can be concluded that by analysing the time required for the chemical homogenization of the steel after the introduction of the alloying additive—calculated for the “pure” marker element—it is possible to conduct calculations for various amounts of additives, ranging from approximately 100 kg to around 500 kg of the marker element. However, it is essential to remember that when determining the homogenization time, additives with a higher mass will be crucial, given that the mass of the metal bath is approximately 103 tonnes (Table 7).

## 3. Results and Discussion

This section presents the results of research using available tools in the field of computational fluid dynamics (CFD), supported by results from research conducted on industrial equipment. The numerical simulations of the process of purging liquid steel with argon were carried out in collaboration with the Chair of Metals Extraction and Recycling of the Częstochowa University of Technology. The tool used to run the simulations was an ANSYS Fluent code.

Based on literature data [17,20,23,24,25,26] and the experience of the research group from the Chair overseeing the numerical studies, a simulation model describing the studied phenomenon was formulated. A two-equation k-ε turbulence model [27,28,29] was used to evaluate the turbulent flow inside the studied ladle furnace, with the standard version with model constants: C_μ_ = 0.09, σ_k_ = 1.0, σ_ε_ = 1.3, C_1ε_ = 1.44, and C_2ε_ = 1.92. Gas bubbles were modelled with the use of the discrete phase model (DPM) [29,30,31] with a uniform diameter distribution of 0.015 m. One-way coupling has been considered with the use of the discrete random walk model for stochastic tracking of particles [32] in order to introduce disturbance in bubble motion. In simulations, all walls of the ladle were considered as stationary walls (boundary condition with u = v = w = 0). It was also assumed that the top surface is a flat free surface—a stationary wall with zero shear stresses (τ_xy_ = τ_xz_ = τ_yz_ = 0). Argon bubbles flow out from the purging plug and are floating in the model fluid, and then they leave the system after reaching the top free surface. A contact of bubbles with other walls of the object causes their reflection. Additionally, some model simplifications were introduced in the numerical studies:

- The influence of slag covering the metal bath was omitted—the metal surface was represented as a flat, free surface.
- The gas-permeable fitting was modelled as a smooth surface, from which argon was released at a specified flow rate in the form of bubbles with a fixed diameter.

**Figure 3:**
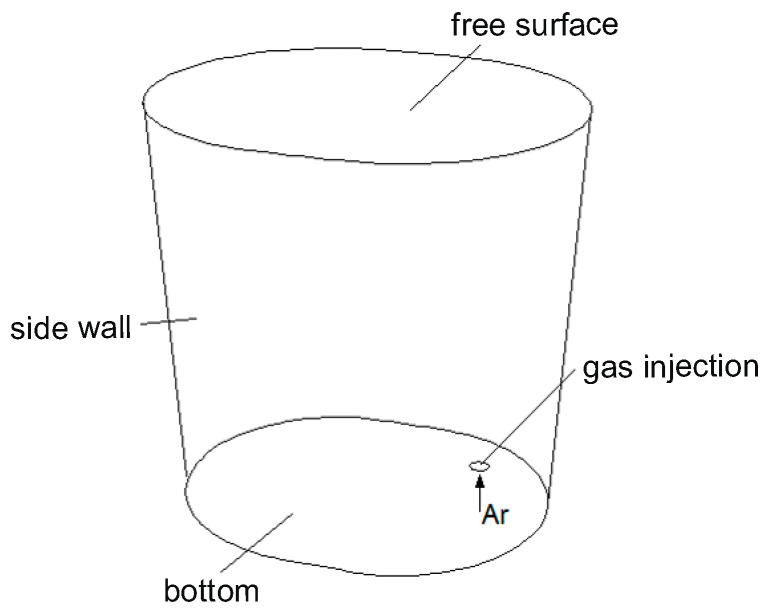
Boundary conditions adopted in the calculations.

Figure 3 illustrates the boundary conditions applied in the numerical simulations. The bottom of the ladle and the sidewall, shown in the figure, were modelled using the stationary wall boundary condition, while the free surface was represented by a wall with zero shear stress. The gas-permeable fitting delivers gas at a preset flow rate. Gas bubbles are removed from the computational domain only upon contact with the free surface; otherwise, they are reflected.

The results of the numerical simulations carried out as part of the paper were compared with experimental data obtained from the industrial object. The industrial experimental research was carried out for two different gas flow rates of, respectively, 300 dm^3^/min and 500 dm^3^/min.

**Figure 4:**
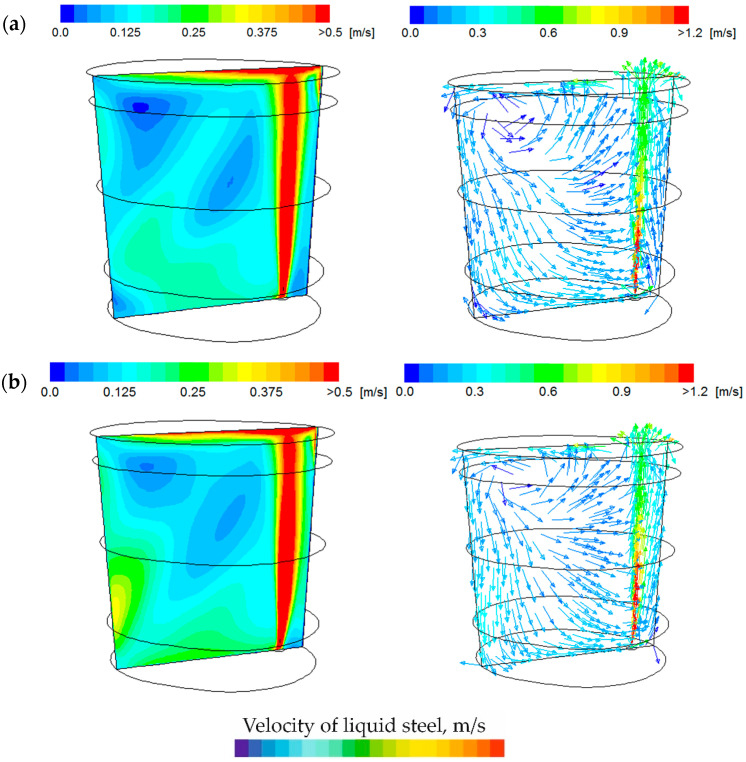
Velocity contour maps and distribution of liquid steel velocity vectors: (**a**) argon flow rate of 300 dm^3^/min; (**b**) argon flow rate of 500 dm^3^/min.

Figure 4 shows the velocity contour maps and the distribution of velocity vectors on the plane passing through the gas-permeable fitting and the ladle axis for the two analysed argon flow rates. By analysing the contour maps, it can be observed that in both cases, a similar pattern of velocity distribution inside the ladle and the locations of flow dead zones are visible. The differences in values are due to the amount of gas supplied by the gas-permeable fitting. However, in both cases, the gas–liquid column determines the movement inside the steelmaking ladle. A characteristic swirling motion is created, where the liquid steel above the gas-permeable fitting is lifted towards the slag phase; then, it dissolves under the slag surface and is directed towards the bottom of the ladle. This flow structure promotes a quicker homogenization of the metal bath, both chemically and thermally.

The aim of the industrial research carried out at the steel plant was to determine the mixing characteristics of the steel bath after the introduction of the alloying additives FeMn and FeSiMn, with the compositions given in Table 3. The treatment in the ladle furnace for the heat options subjected to experimental measurements in both cases involved adjusting the chemical composition after achieving the appropriate steel temperature and level of deoxidation. The amount of additive was determined based on the measurement of the element’s content in the initial sample, and its exact quantity was calculated using suggestion software. After specifying the exact amount of added ferroalloy, it was fed into the bath near the exposed surface of the metal in the ladle, which was made possible by the exposure of the slag by the escaping argon bubbles (at the axis of the gas-permeable fitting). Data regarding the industrial heat options, including the quantities of added ferroalloys and the argon flow rates, are presented in Table 10.

Other activities during the implementation of the experimental determinations involved the method and timing of the collection of subsequent metal samples after the introduction of the marker. The changes in chemical composition over time following the addition of the marker were monitored by sampling at a rate determined by the individual intervals. The sampling location—due to the use of a device integrated with the ladle furnace dome—was consistent and repeatable for all conducted measurements. The sampling frequency was determined for each series of tests. The method of sampling and determination made it possible to use the standard samplers employed at the steel plant, while minimising the measurement errors that could have occurred in the case of manual sampling (such as different sampling locations, particularly varying depths). After cooling, the samples were properly labelled, ground, and then subjected to the spectrometric chemical analysis, during which the chemical composition of the samples was determined with an accuracy to three decimal points. The result is the arithmetic mean of three consecutive correct “sparks” of the determined elements.

Other industrial experiment data are presented in Table 11.

The results of the industrial measurements, in addition to their inherent scientific and exploratory value, were used to verify the numerical model describing the mixing of the metal bath in the ladle furnace after the introduction of alloying additives into the steel and describing the thermal homogenization process of the liquid metal.

**Figure 5:**
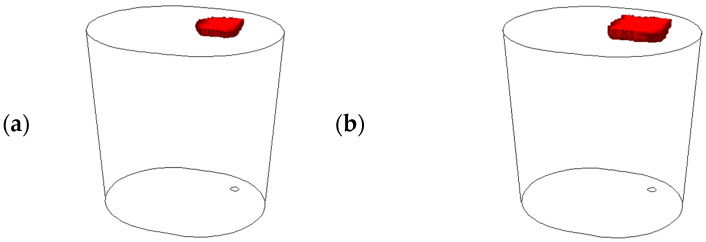
Location of alloying additive: (**a**) FeMn at 200 kg; (**b**) FeSiMn at 502 kg. (red object—alloying additive).

The comparison of the numerical simulation results with the experimental data was based on the analysis of the chemical and thermal homogenization process. As indicated in Table 12, the alloying additive was FeMn at 200 kg and FeSiMn at 502 kg. Figure 5 schematically shows the location of the alloying additive in the simulation models.

In contrast to real-world conditions, the numerical model assumes that the entire mass of the alloying additive is injected all at once and immediately fills a specific area of the computational domain corresponding to the desired mass of the additive. In the numerical simulations conducted, the influence of chemical reactions occurring during the introduction of the alloying additive into the metal bath was omitted. Based on literature data, it is possible to determine the mass of the individual elements contained in the alloying additive involved in the deoxidation process of the steel. For example, the amount of manganese required to change the oxygen content in liquid steel by 0.01% is 0.3 kg/t at a bath temperature of 1568 °C [17]. Taking the above considerations into account, and assuming that only manganese will be subjected to a comparative analysis when studying chemical homogenization, the mass of the alloying additive in the conducted numerical simulations was ultimately converted to the mass of the pure element introduced during the corresponding measurement under industrial conditions.

**Figure 6:**
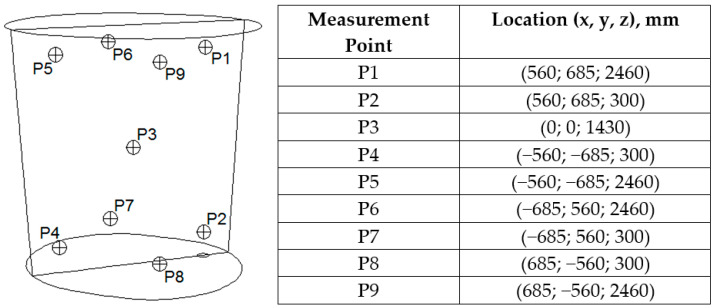
Location of measurement points employed in numerical models.

Figure 6 shows the location of the monitoring points for the concentration of the alloying additive in the numerical simulations, along with exact coordinates, with measurement point P1 corresponding to the location of the concentration monitoring of the alloying additive under industrial conditions.

**Figure 7:**
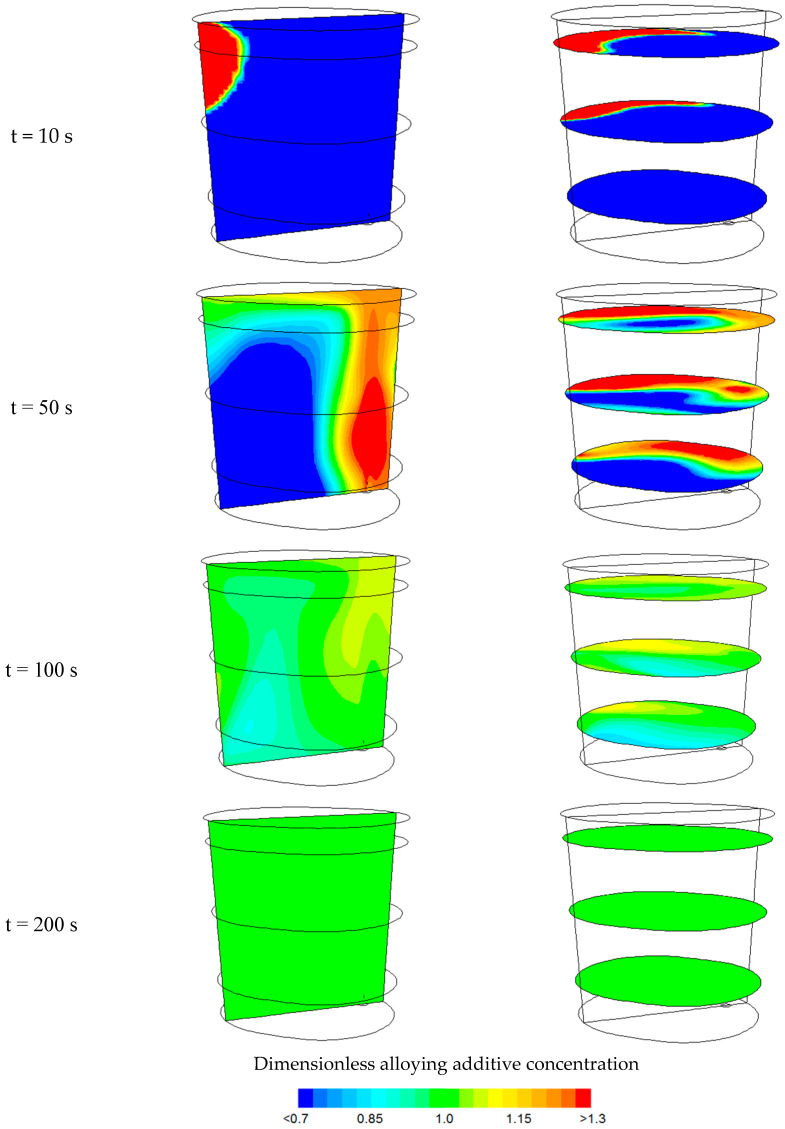
Contour maps of dimensionless alloying additive concentration after the indicated time for an argon flow rate of 300 dm^3^/min.

Figure 7 shows contour maps of the change in dimensionless manganese concentration (alloying additive of 200 kg) after the indicated time for an argon flow rate of 300 dm^3^/min. The contour maps are shown on a vertical plane passing through the centre of the gas-permeable fitting and the axis of the ladle, as well as on horizontal planes located at distances of 0.5 m, 1.5 m, and 2.5 m from the bottom of the ladle.

The dimensionless concentration is defined as

(3) $$C_{Mn}=\frac{C_{t}-C_{0}}{C_{\infty }-C_{0}}$$

where C_t_, C_0_, and C_∞_ represent the concentration of the marker at time t, at the beginning of the process, and at the end of the process, respectively.

Predictions of the state of chemical homogenization, obtained from numerical simulations, show that areas of homogeneous component concentration can only be observed after 100 s.

**Figure 8:**
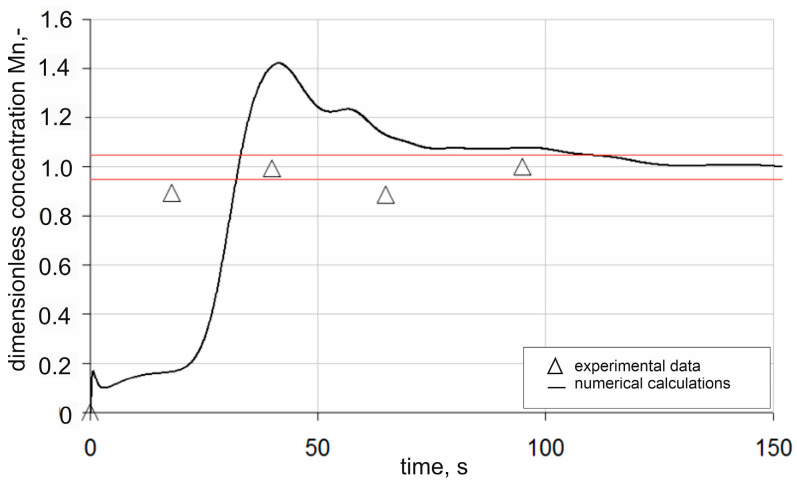
Changes in dimensionless alloying additive concentration in the ladle during argon injection at a rate of 300 dm^3^/min for measurement point P1 (red lines—95% homogenization range).

Figure 8 presents a comparison of the numerical simulation results and experimental data of the dimensionless manganese concentration for measurement point P1. In addition, the graph shows the upper and lower limits of the 95% chemical homogenization band. When analysing the results, it can be seen that in the first time interval, i.e., up to about 75 s after the introduction of the alloying additive, the results of the numerical simulations deviate significantly from the experimental data. The simulation shows a substantial overestimation of the dimensionless concentration of manganese. These differences may also be due to insufficient sampling of the manganese concentration under industrial conditions. As time progresses, beyond 75 s after dosing the alloying additive, the numerical simulation results begin to converge with the data obtained under industrial conditions. Ultimately, based on the results obtained for measurement point P1, it can be concluded that the time required to achieve a homogeneous mixture is approximately 108 s.

For the final verification and determination of the time required to achieve chemical homogenization, data from the other measuring points, P2–P9, were analysed in a manner similar to that of measurement point P1. The maximum time required to reach a 95% homogeneous mixture, as obtained from the numerical simulations, is summarised in Table 12. It follows that measuring point P1 does not indicate the location with the maximum time necessary to achieve a homogenous mixture, and the longest time was recorded at measuring point P4. It is located in the stagnation zone—in the so-called ‘dead’ zone, where bath movement is much less intense than in the rest of the steelmaking ladle.

**Figure 9:**
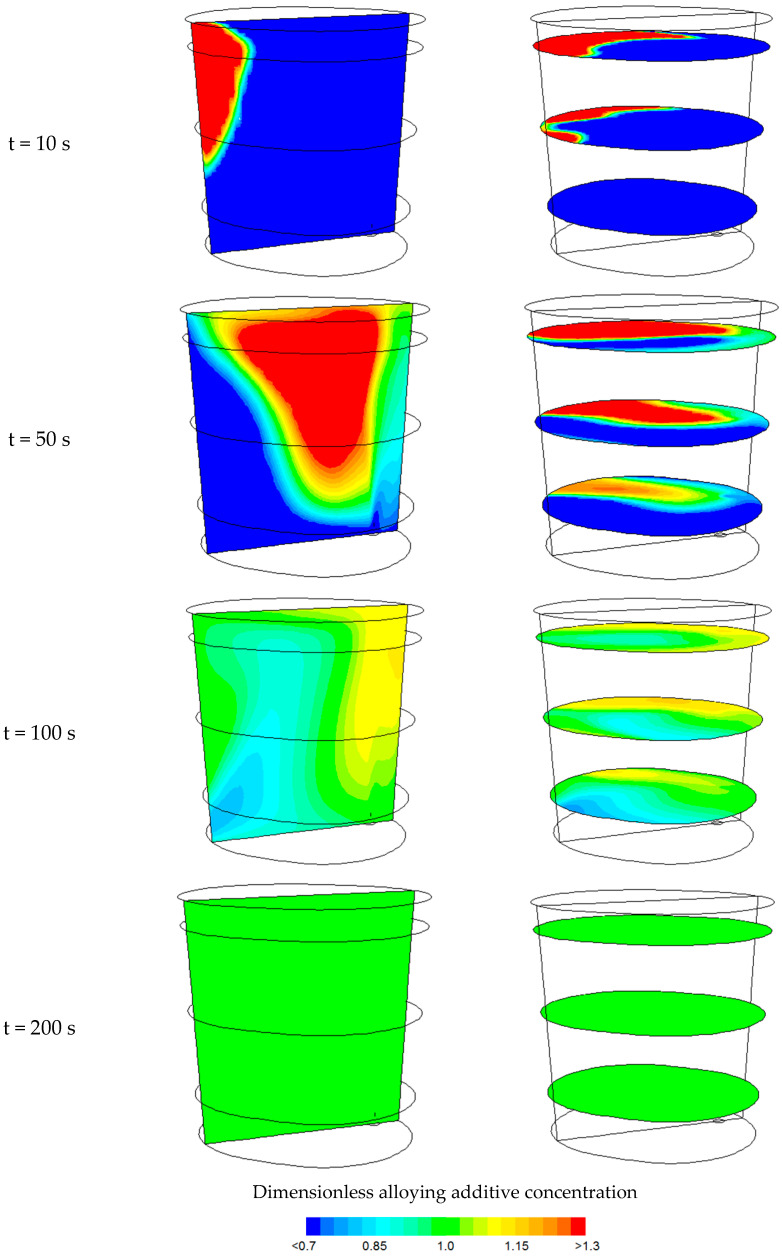
Contour maps of dimensionless alloying additive concentration after the indicated time for an argon flow rate of 500 dm^3^/min.

**Figure 10:**
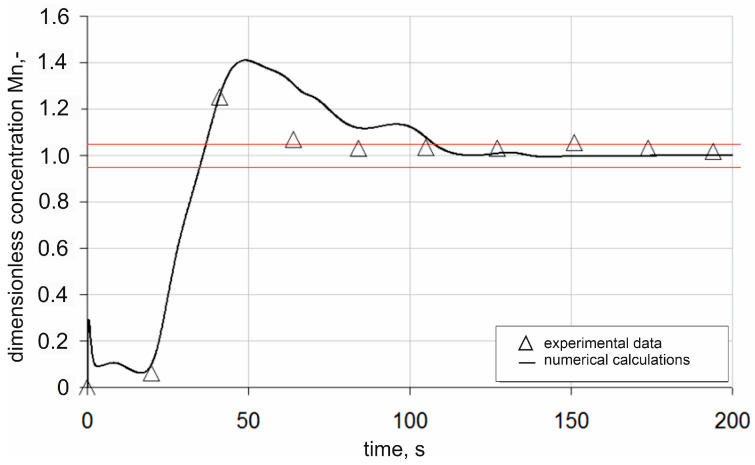
Changes in dimensionless alloying additive concentration in the ladle during argon injection at a rate of 500 dm^3^/min for measurement point P1 red lines—95% homogenization range.

Figure 9 shows contour maps of the change in dimensionless manganese concentration (alloying additive of 502 kg) after the indicated time for an argon flow rate of 500 dm^3^/min. The contour maps are shown on a vertical plane passing through the centre of the gas-permeable fitting and the axis of the ladle, as well as on horizontal planes located at distances of 0.5 m, 1.5 m, and 2.5 m from the bottom of the ladle. The chemical homogenization state predictions obtained from the numerical simulations show that areas of homogeneous component concentration do not appear after 100 s, as was the case with the results described earlier in this paper. The significant increase in the mass of the alloying additive, along with the increased argon flow rate, slightly extended the time required to achieve a homogeneous mixture. Comparing the results of the numerical simulation with the experimental data of the dimensionless manganese concentration for the measurement point P1 (Figure 10), it can be observed that the numerical simulations accurately predict the homogenization process, and its time for the measurement point P1 is approximately 107 s. The analysis of the other measurement points, P2–P9, indicated a maximum homogenization time of 128 s, located at measurement point P7 (Table 13). As in the case analysed in the earlier part of the paper, this point is located in a zone of a reduced flow rate. Analysing the results obtained from the numerical simulations so far, it can be seen that they provide an excellent complement to the experimental data and, when combined, offer a much broader perspective on the phenomenon of chemical homogenization in the studied industrial object.

### 3.1. Predicting Chemical Homogenization Time

Following the validation of the numerical model, it was used to carry out calculations to predict the chemical homogenization time for selected argon flow rates, within the optimum range for the ladle furnace under study, i.e., from 300 to 500 dm^3^/min.

**Figure 11:**
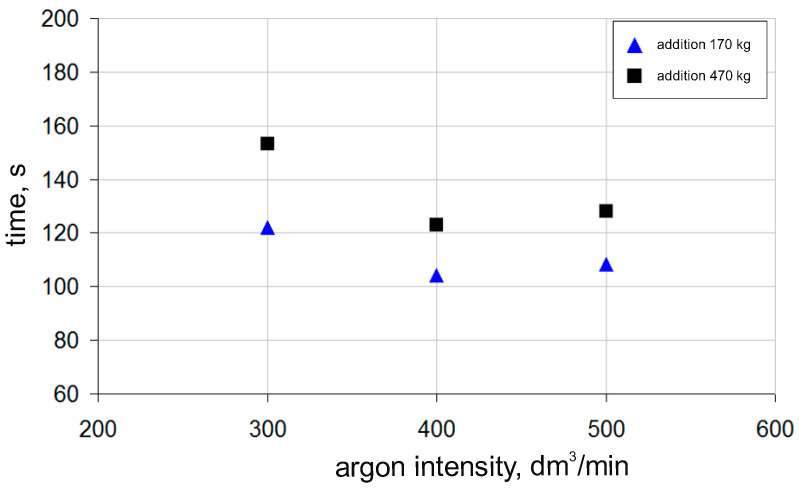
Numerically determined mixing time required to achieve 95% chemical homogenization of metal bath, as function of argon flow rate and mass of alloying additive.

Two alloying additives—one weighing 170 kg and the other 470 kg—were selected as representative mean masses of alloying additives from two groups: smaller and larger alloying additives, respectively, used in industrial practice. The results are presented in Figure 11. Analysing the above data, it can be seen that as the mass of the alloying additive increases, the time required to achieve a homogeneous mixture also increases. Additionally, it can be noted that the optimal time to achieve a homogeneous mixture is obtained—for both cases—at an argon flow rate of 400 dm^3^/min. A higher gas flow rate may slightly extend the chemical homogenization time, which is likely due to the fact that as the flow of the injected gas increases, the turbulence in the bath within the studied industrial object also increases. This relationship was observed for both studied sizes of alloying additives, leading to the conclusion that the optimum argon flow rate for ensuring the rapid achievement of the desired level of the chemical homogenization of the metal bath in the studied ladle is 400 dm^3^/min.

### 3.2. Thermal Homogenization of Metal Bath

As part of the research presented in this paper, numerical simulations of the thermal homogenization of the metal bath following the introduction of an alloying additive at ambient temperature were also carried out.

Simulations of the mixing of the liquid steel after the introduction of the alloying additive, taking into account heat transfer, were carried out to determine whether the final effect on the homogenization of the process is determined by chemical or thermal homogenization. In the simulations, it was assumed that the heat loss fluxes were 12 kW/m^2^ for the free surface, while for the remaining walls, the heat losses were assumed to be at the level of 5 kW/m^2^ [33,34]. Industrial measurements were conducted for a heat with a mass of 106.1 tonnes. The alloying additive consisted of 200 kg of FeMn, and the argon flow rate was 370 dm^3^/min. The temperature was measured immediately after the additive was introduced and after 30, 60, 90, and 120 s, i.e., until the expected time to achieve the chemical homogenization of the metal bath.

**Figure 12:**
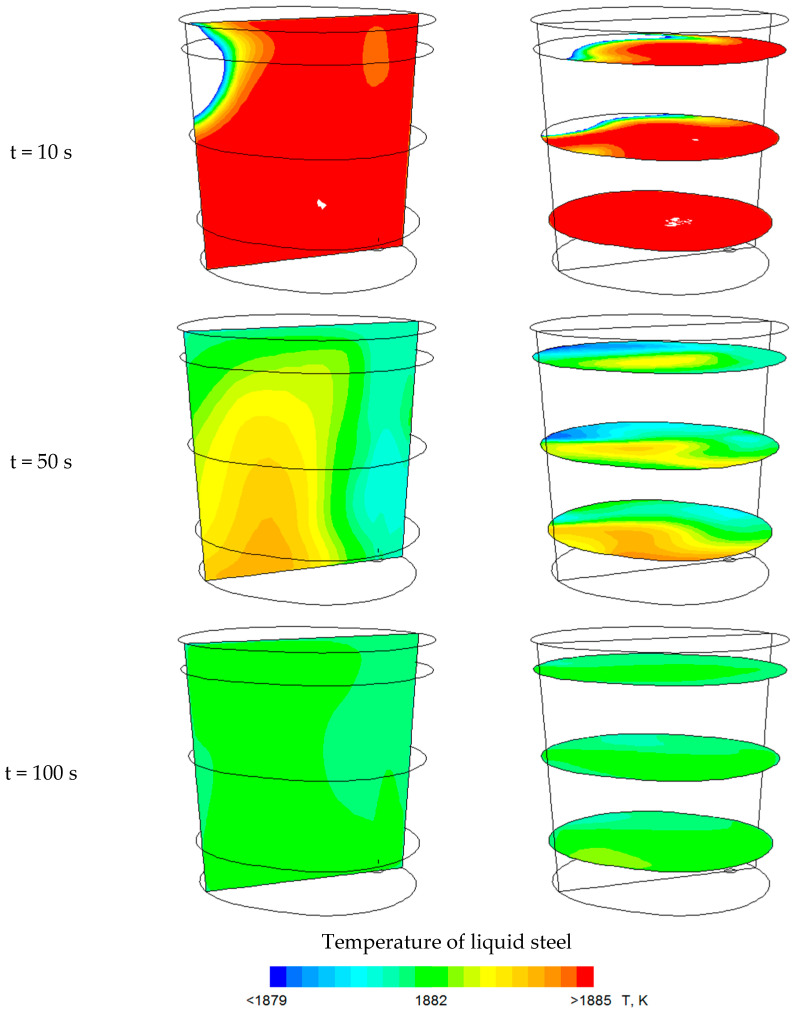
Temperature contour maps after the indicated time, for an argon flow rate of 370 dm^3^/min.

**Figure 13:**
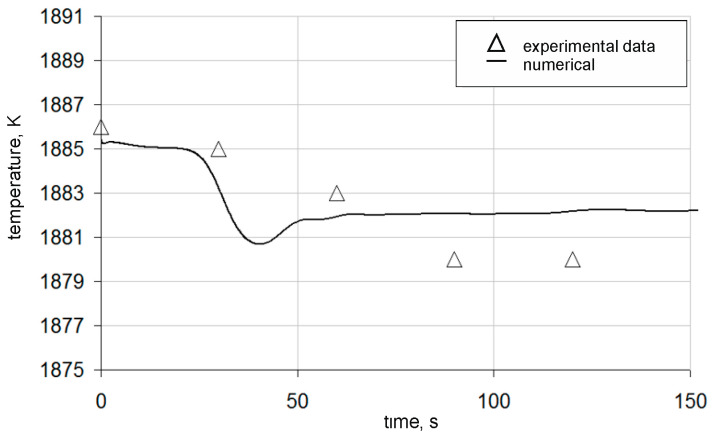
Temperature variations in the ladle during argon injection at a flow rate of 370 dm^3^/min, for the measurement point P1.

Figure 12 presents contour maps of temperature variation after the indicated time for an argon flow rate of 370 dm^3^/min. The contour maps are shown on a vertical plane passing through the centre of the gas-permeable fitting and the axis of the ladle, as well as on horizontal planes located at distances of 0.5 m, 1.5 m, and 2.5 m from the bottom of the ladle. To better represent the temperature changes, the scale was limited to Δ = 6 K. White (blank) areas indicate that the temperature scale was exceeded; however, this is the case only for the contour maps presented for time t = 10 s. The predictions of the state of thermal homogenization obtained in the numerical simulations show that areas with a homogeneous temperature distribution appear after 100 s, with temperature variations of no more than 2 K throughout the computational area. Figure 12 shows a comparison of temperature variations in the ladle for the measurement point P1. When comparing the results, a high level of consistency can be observed between the numerical simulation results and the experimental data, given that the numerical model includes some simplifications, such as the absence of chemical reactions occurring in the metal bath (Figure 13). Nevertheless, the time to achieve thermal homogenization for the results of the numerical simulations and the experimental data is similar, and with regard to chemical homogenization, it is much shorter. Therefore, it can be stated that thermal homogenization occurs earlier, and the entire homogenization process is completed when the chemical homogenization process is concluded.

The analysis of the results from numerical studies and the verifying industrial measurements of the thermal homogenization process of steel in a ladle furnace showed that the time required to achieve a temperature-homogeneous liquid metal, from the moment the alloying additive is introduced into the ladle furnace, is approximately 110 s.

## 4. Conclusions

The results of the industrial experimental research and numerical studies presented in this paper led to the following statements and conclusions:

1. Numerical modelling makes it possible to determine the mixing characteristics of the alloying additive in the steelmaking ladle and to determine the time required to achieve the assumed degree of the chemical homogenization of the metal bath. Due to certain simplifications in the numerical model, it is not possible to determine the actual characteristics of changes in marker concentration at the monitoring point corresponding to the metal sampling point in the industrial object (P1). Nevertheless, the numerically calculated mixing time (for all designated monitoring points) coincides with the time obtained experimentally. One has to keep in mind that numerical simulations have some simplification; therefore, results supported by industrial measurements are more valuable.
2. At the same time, it was shown that point P1 (where samples are collected from the actual device) is not adequate for determining chemical homogenization throughout the entire ladle space. Nevertheless, the differences in the degree of chemical homogenization between the recorded measurement points in the ladle space, as determined by numerical studies, are small enough to be negligible for industrial practice.
3. Purging the liquid steel with argon gas (so-called argon purging) at an appropriate rate enables the temperature and chemical composition of the metal bath to be equalised relatively quickly (in relation to the duration of the process) after the introduction of the alloying additive.
4. The results of the research clearly demonstrate that the thermal homogenization time of the liquid metal, after the introduction of the alloying additive, is shorter than its chemical homogenization time. Therefore, it can be concluded that the homogenization time of the metal bath is determined by its chemical homogenization.
5. A potential area of future investigations is using another porous plug. The industrial ladle is equipped with two porous plugs. In normal conditions, an eccentric porous plug is used (no. 1), but it is possible to use a second porous plug (named “central”, no. 2) as well. Future investigation can be performed with using a second plug as support.

## Figures and Tables

**Table 1:** Basic data of the ladle furnace.

Supplier	ELKEM—SCANLANCE—SWEDEN
Year of manufacture	1993
Estimated treatment time	45 min (excluding steel transport time)
Amount of steel processed per batch	100 tonnes
Transformer power	30 MVA
Graphite electrodes	three electrode columns
Electrode diameter	406 mm
Electrode pitch diameter	640 mm

**Table 2:** Chemical composition of S355 steel (%mass, ppm).

	C	Mn	Si	P	S	Cr	Ni	Cu	Mo	Al._tot_
min.	0.14	1.36	0.20	-	-	-	-	-	-	0.020
max.	0.17	1.45	0.50	0.020	0.020	0.20	0.30	0.30	0.080	-

**Table 3:** Chemical composition of FeMn used in industrial measurements.

	Mn	C	Si	P	S
FeMn% _mass_	69.6	4.3	1.6	0.02	0.018
FeSiMn% _mass_	66.6	1.46	18.1	0.25	0.027

**Table 4:** Temperatures recorded during industrial measurements.

Subsequent Number	Temperature, °C	
	Entry	Exit
1	1530	1554
2	1540	1555
3	1542	1553
4	1564	1566
5	1542	1556
6	1542	1556
7	1536	1556
8	1557	1563
9	1554	1558
10	1526	1556
11	1525	1560
12	1570	1563
13	1538	1561
14	1534	1558
15	1547	1561
16	1540	1559
17	1533	1562
18	1539	1559
19	1518	1558
20	1526	1561
21	1524	1562
22	1537	1562
23	1525	1562
24	1515	1560
25	1556	1562
26	1527	1568
27	1544	1563
28	1531	1555
mean:	1538	1560

**Table 5:** Calculated steel density, for extreme C contents (in % mass) in steel.

C, % Mass	Temperature, °C	Density, kg/m^3^
0.14	1560	7027
0.17		7029

**Table 6:** Values of physico-chemical parameters of liquid steel in the ladle process.

Parameter	Symbol	Units	Value
Specific heat of steel	c_p_	J/kg·K	680 *
Thermal conductivity coefficient of steel	k	W/m·K	40 *
Dynamic viscosity of steel	μ	kg/m·s	0.0062 *

* Values adopted from [14,15].

**Table 7:** Smelting data recorded during treatment at the LF.

Subsequent Heat Number	Treatment Time, min	Argon Quantity, m^3^	Average Argon Flow, dm^3^/min	Mass of Steel, Tonne
1.	107	30.3	283	99.9
2.	31	7.2	232	87.1
3.	29	9.0	310	99.9
4.	39	16.9	433	86.1
5.	23	2.3	100	100.2
6.	23	13.3	578	98.6
7.	30	9.4	313	96.8
8.	25	12.2	488	100.2
9.	97	16.1	166	101.3
10.	34	5.7	168	109.0
11.	39	15.0	385	105.5
12.	34	6.2	182	102.1
13.	43	14.5	337	102.9
14.	41	15.7	383	104.5
15.	39	17.2	441	104.3
16.	34	15.1	444	99.4
17.	39	16.4	421	104.8
18.	29	10.8	372	101.4
19.	25	13.8	552	107.6
20.	31	11.5	371	104.7
21.	23	14.4	626	109.7
22.	102	28	275	108.3
23.	37	19.1	516	104.4
24.	31	16.9	545	104.0
25.	32	14.6	456	100.2
26.	90	19.8	220	110.2
27.	39	5.9	151	102.4
28.	45	32.9	731	99.6
29.	38	15.8	416	104.3
30.	36	19.2	533	106.6
31.	36	21.2	589	100.5
32.	90	15.1	168	111.0
33.	38	12.1	318	102.6
34.	33	12.3	373	101.6
35.	61	11.3	185	104.9
36.	25	7.9	316	104.8
37.	27	9.6	356	108.1
38.	26	7.7	296	104.3
39.	55	14.2	258	103.4
40.	30	7.3	243	106.3
41.	34	5.9	174	102.6
42.	35	9.3	266	105.1
43.	39	11	282	104.7
44.	33	12	364	105.2
45.	35	12.2	349	106.4
46.	39	10.1	259	106.9
Mean value:				103.1

**Table 8:** Critical argon flow rate that prevents slag interruption.

Bath Height	H	Cm	283			
Steel–slag interfacial tension *	σ	dyne/cm	1137	1050	1050	1050
Slag density	ρ_slag_	g/cm^3^	2.5	2.5	2.7	2.5
Steel density	ρ_s_	g/cm^3^	6.9	6.9	7.1	7.1
Density difference	Δr	g/cm^3^	4.4	4.4	4.4	4.6
Critical flow rate	Q_cr_	dm^3^/min	597	581	550	590

* Values adopted from [17,18,19,20,21,22].

**Table 9:** Additives introduced during treatment at the LF station.

Subsequent Heat Number	Alloying Additives—Type/Quantity, kg		
	FeMn	FeSiMn	FeSi
1.	74	-	-
2.	214	-	-
3.	74	300	172
4.	100	454	52
5.	106	-	-
6.	188	-	50
7.	192	202	-
8.	136	-	-
9.	24	-	-
10.	-	260	154
11.	232	200	252
12.	-	300	-
13.	260	-	-
14.	-	236	182
15.	FeNb = 36	474	40
16.	-	254	274
17.	FeNb = 42	-	-
18.	134	-	-
19.	426	-	52
20.	260	-	62
21.	190	80	152
22.	46		-
23.	-	-	52
24.	180	-	-
25.	198	-	32
26.	142	200	-
27.	54	-	32
28.	46	-	-

**Table 10:** Data of recorded industrial heat options.

Designation	Heat	Type of Marker	Material Carrying the Marker	Mass of Ferroalloy [kg]	Standard Argon Flow Rate [dm^3^/min]
B0–B4	582,291	Mn	FeMn	200	300
A0–A12	581,981	Mn	FeSiMn	502	500

**Table 11:** Other industrial experiment data.

Designation	Heat	Heat Mass [Tonnes]	Sampling Frequency	Comments
B0–B4	582,291	104.3	sample/23 s	a new sample was collected every 23 s throughout the measurements
A0–A12	581,981	106.4	sample/21 s	a new sample was collected every 21 s throughout the measurements

**Table 12:** Predicted time for chemical homogenization of bath (300 dm^3^/min).

Variant	The 95% Chemical Homogenization of the Bath in the Selected Ladle Monitoring Locations [s]:								
	P1	P2	P3	P4	P5	P6	P7	P8	P9
300 dm^3^/min	108.7	87	90.2	122	98.6	112.2	107.9	114.1	88.3

**Table 13:** Predicted time for chemical homogenization of bath (500 dm^3^/min).

Variant	The 95% Chemical Homogenization of the Bath in the Selected Ladle Monitoring Locations [s]:								
	P1	P2	P3	P4	P5	P6	P7	P8	P9
500 dm^3^/min	107.3	94.5	115.1	112.8	102.2	105.8	128	117.2	85.6

## Data Availability

The original contributions presented in this study are included in the article. Further inquiries can be directed to the corresponding author.
